# Implementing Experience Sampling Technology for Functional Analysis in Family Medicine – A Design Thinking Approach

**DOI:** 10.3389/fpsyg.2019.02782

**Published:** 2019-12-11

**Authors:** Naomi E. M. Daniëls, Laura M. J. Hochstenbach, Marloes A. van Bokhoven, Anna J. H. M. Beurskens, Philippe A. E. G. Delespaul

**Affiliations:** ^1^Department of Psychiatry and Neuropsychology, School for Mental Health and Neuroscience, Maastricht University, Maastricht, Netherlands; ^2^Department of Family Medicine, Care and Public Health Research Institute, Maastricht University, Maastricht, Netherlands; ^3^Research Centre for Remote Health Care, Zuyd University of Applied Sciences, Heerlen, Netherlands; ^4^Research Centre for Autonomy and Participation for Persons with a Chronic Illness, Zuyd University of Applied Sciences, Heerlen, Netherlands; ^5^Mondriaan Mental Health Trust, Heerlen, Netherlands

**Keywords:** development, ecological momentary assessment, Experience Sampling Method, mHealth, family medicine, redesign, self-monitoring, telemedicine

## Abstract

**Background:**

A paradigm shift in health care from illness to wellbeing requires new assessment technologies and intervention strategies. Self-monitoring tools based on the Experience Sampling Method (ESM) might provide a solution. They enable patients to monitor both vulnerability and resilience in daily life. Although ESM solutions are extensively used in research, a translation from science into daily clinical practice is needed.

**Objective:**

To investigate the redesign process of an existing platform for ESM data collection for detailed functional analysis and disease management used by psychological assistants to the general practitioner (PAGPs) in family medicine.

**Methods:**

The experience-sampling platform was reconceptualized according to the design thinking framework in three phases. PAGPs were closely involved in co-creation sessions. In the ‘understand’ phase, knowledge about end-users’ characteristics and current eHealth use was collected (nominal group technique – 2 sessions with *N* = 15). In the ‘explore’ phase, the key needs concerning the platform content and functionalities were evaluated and prioritized (empathy mapping – 1 session with *N* = 5, moderated user testing – 1 session with *N* = 4). In the ‘materialize’ phase, the adjusted version of the platform was tested in daily clinical practice (4 months with *N* = 4). The whole process was extensively logged, analyzed using content analysis, and discussed with an interprofessional project group.

**Results:**

In the ‘understand’ phase, PAGPs emphasized the variability in symptoms reported by patients. Therefore, moment-to-moment assessment of mood and behavior in a daily life context could be valuable. In the ‘explore’ phase, (motivational) functionalities, technological performance and instructions turned out to be important user requirements and could be improved. In the ‘materialize’ phase, PAGPs encountered barriers to implement the experience-sampling platform. They were insufficiently facilitated by the regional primary care group and general practitioners.

**Conclusion:**

The redesign process in co-creation yielded meaningful insights into the needs, desires and daily routines in family medicine. Severe barriers were encountered related to the use and uptake of the experience-sampling platform in settings where health care professionals lack the time, knowledge and skills. Future research should focus on the applicability of this platform in family medicine and incorporate patient experiences.

## Introduction

Modern health care is faced with two challenges: refocus from an overemphasis on illness to wellbeing to better realize the multidimensional experience of positive health; and the design and implementation of new technologies that support this process. The diagnosis evidence-based group-level symptom-reduction model is under strain because it ignores the patient’s needs and goals, the transdiagnostic psychological mechanisms at stake in mental disorders, and the importance of the therapist–patient relationship in care (van Os et al., 2019). Mental illness is better framed as vulnerabilities. Consequently, patients learn how to cope with these vulnerabilities and develop resilience to increase their wellbeing. The shift from illness to wellbeing is central in the Dutch New Mental Health Movement. It links the concept of recovery to positive health and proposes personalized psychiatry to reach its goals. The vision, developed by the Dutch New Mental Health Movement, states that mental health is contextualized and consequently care should occur embedded within the community. This newly developed vision leads to three engagements: patient engagement according to ‘nothing about us without us,’ shifting resources from clinical facilities to the neighborhood (context involvement), and enriching contacts with network-based care, both face-to-face and through an online community (virtual expansion) (Delespaul et al., 2017). Care should be a collaborative process between health care professionals, patients and meaningful persons in their environment. In this process, patients should stay in control of their own care process and get empowered to actively participate in their treatment. Equally so, their supportive environment should also be empowered to realize sustainable solutions. These developments are in line with the new definition of health, “as the ability to adapt and control your own life, in the light of social, mental and physical challenges in life,” proposed in alternative to the WHO definition of health that was criticized because it tended to medicalize all deviations of optimal functioning (Huber et al., 2011). Health is defined in relation to personal goals. It requires a personalized psychiatry which takes into account patients’ characteristics, needs and desires in their care process (Perna et al., 2018). These developments create a paradigm shift from illness to wellbeing.

New technologies can support this process, but implementation and adoption are challenging. Previous literature shows that only a few studies about the implementation of eHealth in practice are available. They show that eHealth tools are often poorly implemented (Rabin and Glasgow, 2012; Swinkels et al., 2018). An essential step in the successful implementation of eHealth is the active involvement of end-users during the development process (van Gemert-Pijnen et al., 2011; van Bruinessen et al., 2014; de Beurs et al., 2017; Swinkels et al., 2018). They focus on the realities of daily practice where eHealth solutions often are less usable and feasible (van Gemert-Pijnen et al., 2011; Swinkels et al., 2018). It is essential to take these requirements into consideration to bridge the gap between tools used in research and tools used in daily clinical practice.

Relying on eHealth to realize the paradigm shift from illness to wellbeing is not straightforward and requires a fundamentally new approach. The reason is that eHealth tools are often modified classic interventions that focus on symptom reduction using the strategy of learning skills that should generalize in daily situations. Although anxious patients can learn to relax during (classic) therapy or even in front of a computer at home (eHealth), it is possible that they panic in daily life situations. Transfer from the therapist’s office to the individual’s daily life cannot be taken for granted. Consequently, self-monitoring tools based on the Experience Sampling Method (ESM) are proposed. Data is collected in daily life using (random) time-based sampling triggers that generate assessments of vulnerable as well as resilient moments. This well-designed data collection procedure with customizable questionnaires is well-accepted in diverse populations (Heron and Smyth, 2010; Myin-Germeys et al., 2018). In ESM, individuals are asked to complete, for example, a short 2-min questionnaire about thoughts, mood and context at several moments a day and for several days in response to sound triggers (beeps) (Larson and Csikszentmihalyi, 1983). This gives insight into daily life functioning (Larson and Csikszentmihalyi, 1983). This method has previously been applied in specialized mental health (Delespaul et al., 2002; Kramer et al., 2014; Simons et al., 2015), but it has never been studied as a data collection method in family medicine where patients present with different problems. This requires reappraisal of methods and procedures. A new setting, a new target population and a new goal of the data collection method ask for a translation from science into daily clinical practice.

Therefore, the main aim of the paper is to investigate the redesign process of the PsyMate^TM^, an ESM tool for detailed functional analysis and disease management, for use by psychological assistants to the general practitioner in family medicine. A more thorough insight into the redesign process of the PsyMate^TM^ in family medicine is needed to provide practical support for implementation of an ESM tool in family medicine. The following research questions were formulated:

(1) What are the end-users’ characteristics, needs and goals for use of the PsyMate^TM^ in daily clinical practice?
(2) What are the user requirements and how can the PsyMate^TM^ be optimized?
(3) What are the experiences of the psychological assistants to the general practitioner with the redesigned PsyMate^TM^?

## Materials and Methods

### Design

**FIGURE 1:**
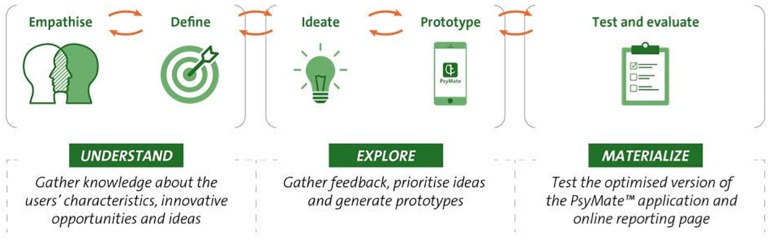
The design thinking model used for the redesign of the PsyMate^TM^.

The model used for the redesign of the PsyMate^TM^ consists of three phases of design thinking (Figure 1) (Gibbons, 2016). The ‘understand’ phase belongs to the concept of understanding the user and the user’s problems and consists of the empathize and define stages. The ‘explore’ phase belongs to the concept of exploring new ideas and representations and consists of the ideate and prototype stages. The ‘materialize’ phase belongs to the concept of materializing these new ideas and representations, and consists of the test and implement stages. In this study, only the test stage was performed. Table 1 provides an overview of the research methodology that was applied in each phase of the design thinking model.

**TABLE 1:** Overview of the goal, participants, procedure and data collection methods, data analysis and results in each phase of the design thinking model.

**Phase**	**Understand**	**Explore**		**Materialize**
			
**Stage**	**Empathize and Define**	**Ideate**	**Prototype**	**Test**
Goal	Explore the characteristics and mental health problems within the patient group (patients consulting a psychological assistant to the general practitioner in family medicine) and the current use of eHealth in daily clinical practice	Collect key needs and suggestions about the content and functionalities of the PsyMate^TM^ application and web-based reporting tool	Build a new prototype based on the ideas	Evaluate the newly developed prototype, i.e., the adjusted version of the PsyMate^TM^ application and web-based reporting tool: barriers and facilitators of use
Research concept	Usability	Usability		Feasibility
Participants	Psychological assistants to the general practitioner (*N* = 30; 2 groups of 15 participants each) Mental health care manager and team leader	Psychological assistants to the general practitioner (*N* = 5) Mental health care manager and team leader Interprofessional project group	Technicians with expertise in software development and design	Psychological assistants to the general practitioner (*N* = 4)
Procedure and data collection methods	Literature scan concerning users’ characteristics and eHealth in clinical care [e.g., purposes, approaches, (dis)advantages, challenges, and use, attitudes and acceptance in family medicine]. Two co-creation sessions with the psychological assistants to the general practitioner, the mental health care manager and the team leader using the nominal group technique concerning the patient group and the current use of eHealth in family medicine (30 min).	One co-creation session with the psychological assistants to the general practitioner and the team leader using empathy mapping with a patient with a lifestyle problem as a use case, collecting key needs and suggestions concerning the content and functionalities of the PsyMate^TM^ application (1 h). One co-creation session with the psychological assistants to the general practitioner and the team leader using moderated user testing, collecting key needs and suggestions concerning the content and functionalities of the PsyMate^TM^ web-based reporting tool (1 h). One co-creation session with the interprofessional project group using the MoSCoW method, prioritizing the collected key needs and suggestions concerning the functionalities of the PsyMate^TM^ application and web-based reporting tool (2 h).		Individual coaching on the job with the psychological assistants to the general practitioner concerning the use of the PsyMate^TM^ in family medicine (30 min). Using the PsyMate^TM^ in daily family medicine practice for 4 months. Focus group with the psychological assistants to the general practitioner concerning the use of the PsyMate^TM^ in family medicine (2 h). Individual semi-structured interviews with the psychological assistants to the general practitioner concerning the use of the PsyMate^TM^ in family medicine (30 min). Three weekly telephone contact with the psychological assistants to the general practitioner concerning the use of the PsyMate^TM^ in family medicine.
Data analysis	Descriptive content analysis	Conventional content analysis		Consolidated Framework for Implementation Research (CFIR; Damschroder et al., 2009)
Results	Patient characteristics: psychosocial problems ranging from symptoms such as sleep and self-esteem problems to disorders such as anxiety and depressive disorders. Current use of eHealth: diagnostic, psycho-educative and intervention purposes.	Key needs: clear and concise items, possibility to add personal items, and an intuitive and easy-to-use tool with a feedback mechanism (visualize the data). Must Haves for the PsyMate^TM^ application: add reward gamification elements, create insight into the progress of the beeps (in-app feedback), develop an explanimation or a manual, and create a personal profile. Must Haves for the PsyMate^TM^ web-based reporting tool: develop a manual, add a reset button and create a simple and advanced web-based reporting tool. Should Haves for the PsyMate^TM^ application: add a memo button. Should Haves for the PsyMate^TM^ web-based reporting tool: make the completed memos visible.	Medium-fidelity prototype of the PsyMate^TM^ application and web-based reporting tool. Adopted changes: option to (de)activate five personal items and option to silence the beep sound for a required time span.	Barriers on the level of the inner setting: limited time per consultation and no commitment of the general practitioners. Barriers on the level of the intervention: no in-app feedback, insufficient instructions and no gamification. Barriers on the level of the individual characteristics: mind shift in the way of working and embedding the PsyMate^TM^ in their working process. Strategies on the level of the process: concrete work instructions by means of a manual and an instruction card, use cases (examples), frequent telephone and mailing contact with a researcher and a WhatsApp group consisting of the psychological assistants to the general practitioner, their team leader and a researcher.

### Setting and Participants

This study was conducted in general practices connected to a regional primary care group in the south of the Netherlands. Psychological assistants to the general practitioner (PAGPs), psychological wellbeing practitioners, have a background as a nurse or doctor’s assistant with an additional 2-years training at a University of Applied Sciences (van Hassel et al., 2016). They work independently, though under supervision of a general practitioner. PAGPs assess the nature and severity of complaints, and determine whether an intervention or referral is needed. Furthermore, their tasks include providing support, guidance and short-term treatment (i.e., consultation, psycho-education, case management, screening diagnostics, self-management and aftercare) for patients with psychological or psychosocial problems.

All PAGPs (*N* = 30) of the regional primary care group were invited and took part in the ‘understand’ phase. They were interested in innovation in family medicine, had at least 2 years of experience and had a full understanding of the Dutch language. Using convenience sampling, PAGPs with an interest in eHealth were recruited via their team leader to take part in the ‘explore’ phase. Five PAGPs (4 women, 1 man) were included. They had a background in social psychiatry, nursing and psychology, worked in two to five general practices and had working experience between 2 and 4 years (*M* = 3.60, *SD* = 0.80). All PAGPs worked with patients suffering from psychosocial problems ranging from depressive and anxiety disorders to eating disorders and trauma. Four of them were engaged in the ‘materialize’ phase as well. In order to process all the input and output during the design thinking process, an interprofessional project group with nine researchers and three technicians from different backgrounds (innovations in mental health, autonomy and participation, efficient monitoring, mental health, eHealth and self-management, public health and primary care, communication and multimedia design, and software design and development) was involved. In addition, the PAGPs were asked to include patients with psychological or psychosocial problems for the patient interviews. Ethical approval was obtained for the patient interviews from the Medical Ethics Review Committee of Zuyderland and Zuyd University of Applied Sciences. Furthermore, we adhered to the approved ethical (consent) procedures of the Medical Ethics Review Committee of Zuyderland and Zuyd University of Applied Sciences. Since the PAGPs were the research participants and patients received regular care, these procedures do not require written informed consent. However, oral informed consent was obtained from both the PAGPs and the regional primary care group. Moreover, they also provided consent for this research via mail. In addition, ethical principles that are outlined in the Dutch “Medical Research Involving Human Subjects Act” were followed throughout the redesign process.

### Intervention

**FIGURE 2:**
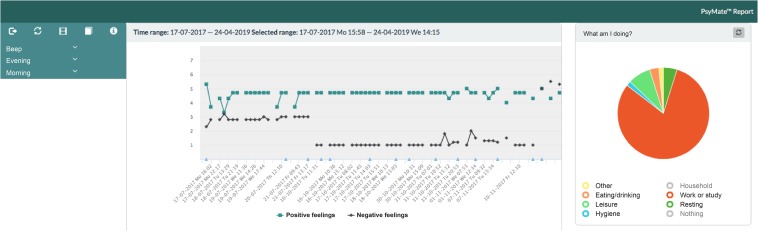
An example of the web-based reporting tool.

PsyMate^TM^ is an ESM platform for moment-to-moment assessment of mood and behavior in the context of daily life and developed by the department of Psychiatry and Neuropsychology of Maastricht University Medical Centre (MUMC+) and Maastricht University (UM)^1^. It consists of a smartphone application (Android and iOS), a cloud-based data system and a web-based reporting tool. The smartphone application generates a beep signal 8–10 times a day at semi-random moments between 7.30 AM and 10.30 PM. Users are requested by beep signals to complete a short questionnaire. Typically, nine mood (i.e., positive and negative affect), three physical status (i.e., hunger, fatigue, pain) and three context (i.e., location, activity and persons present) items are assessed repeatedly. The mood and physical status items are scored on a 7-point Likert Scale (1 = not at all, 4 = moderate, 7 = very) and the context items are assessed categorically. In addition, users are asked to complete a morning questionnaire including four items about sleep quality and an evening questionnaire including five items about the overall appraisal of the day and the subjective experience concerning the use of the PsyMate^TM^. Three items of the morning questionnaire are assessed categorically and one item – overall sleep quality – is rated on a 7-point Likert Scale (1 = not at all, 4 = moderate, 7 = very). All items of the evening questionnaire are rated on a 7-point Likert Scale (1 = not at all, 4 = moderate, 7 = very). Table 2 presents an overview of the beep, morning and evening questionnaires. The responses are immediately available online and displayed graphically in an interactive web-based reporting tool. The Likert scales (mood and physical status beep items, overall sleep quality and end-of-day assessments) yield ordinal scores and are displayed in line charts. The beep-level context items are nominal scales and are depicted in pie charts. This allows patients and health care professionals to make an accurate analysis of specific behavior in the specific context and get insight into behavioral patterns (both related to vulnerability and resilience). Figure 2 illustrates the web-based reporting tool. A detailed description about the tool can be found in Appendix A. All appropriate permissions have been obtained from the trademark holder of PsyMate^TM^ for its use in this research and manuscript.

**TABLE 2:** Beep, morning and evening questionnaire respectively from the PsyMate^TM^ standard assessment protocol.

**Beep questionnaire**	

**Item**	**7-point Likert scale or categorical options**
I feel cheerful	1 = not at all, 4 = moderate, 7 = very
I feel insecure	1 = not at all, 4 = moderate, 7 = very
I feel relaxed	1 = not at all, 4 = moderate, 7 = very
I feel annoyed	1 = not at all, 4 = moderate, 7 = very
I feel satisfied	1 = not at all, 4 = moderate, 7 = very
I feel lonely	1 = not at all, 4 = moderate, 7 = very
I feel anxious	1 = not at all, 4 = moderate, 7 = very
I feel down	1 = not at all, 4 = moderate, 7 = very
I feel guilty	1 = not at all, 4 = moderate, 7 = very
Optional additional personal question	1 = not at all, 4 = moderate, 7 = very
What am I doing?	Select: resting, work or study, household, hygiene, eating/drinking, leisure, other, nothing
I would rather be doing something else	1 = not at all, 4 = moderate, 7 = very
Where am I?	Select: at home, at family or friend’s place, at work or school, public place, transport, somewhere else
Who am I with?	Select: partner, family resident, family non-resident, friends, colleagues, acquaintances, strangers or others, nobody
I am hungry	1 = not at all, 4 = moderate, 7 = very
I am tired	1 = not at all, 4 = moderate, 7 = very
I am in pain	1 = not at all, 4 = moderate, 7 = very
Thank you!	
**Morning questionnaire**	
How long did it take before I fell asleep last night?	Select: 0 – 5 min, 5 – 15 min, 15 – 30 min, 30 – 45 min, 45 min – 1 h, 1 – 2 h, 2 – 4 h, >4 h
How often did I wake up last night?	Select: 0, 1, 2, 3, 4, 5, >5
How long did I lie awake this morning before getting up?	Select: 0 – 5 min, 5 – 15 min, 15 – 30 min, 30 – 45 min, 45 min – 1 h, 1 – 2 h, 2 – 4 h, >4 h
I slept well	1 = not at all 4 = moderate 7 = very much
Thank you!	
**Evening questionnaire**	
I generally felt well today	1 = not at all 4 = moderate 7 = very much
I generally felt tired today	1 = not at all 4 = moderate 7 = very much
I generally felt tense today	1 = not at all 4 = moderate 7 = very much
Filling in the PsyMate^TM^ has influenced my mood	1 = not at all 4 = moderate 7 = very much
Without the PsyMate^TM^ I would have done other things today	1 = not at all 4 = moderate 7 = very much
Thank you!	

### Data Collection and Analysis

#### Phase I: Understand the User and the User’s Problems

In the ‘understand’ phase, the patient characteristics and the mental health problems of patients visiting a PAGP in family medicine, and the current use of eHealth in family medicine were explored.

An exploratory unsystematic review was performed to gather more insight into the organization of mental health care in family medicine, more specifically the purposes, approaches, (dis)advantages, challenges, attitudes and acceptance of the use of eHealth. Outcomes were discussed within the interprofessional project group and assimilated in the next phase. Furthermore, two co-creation sessions with fifteen PAGPs each, their team leader and the mental health care manager were performed using the nominal group technique (Deip et al., 1977) to explore the patient group (patient characteristics and symptoms) and the current use of eHealth. The PAGPs were asked to write down for themselves how they would characterize the patients with mental health problems in their practice. Afterward, each participant provided one patient characteristic in turn without any discussion. Finally, these characteristics were ranked by importance. The same procedure was performed for the eHealth applications they use in the diagnosis and treatment of these patients. Field notes were recorded and analyzed by means of descriptive content analysis. A first list of user requirements was compiled.

#### Phase II: Explore New Ideas and Representations

In the ‘explore’ phase, the PAGPs reported on the key needs regarding the content and functionalities of the PsyMate^TM^ application and web-based reporting tool. Furthermore, they provided suggestions for further optimization of the PsyMate^TM^ application and web-based reporting tool. Moreover, the interprofessional project group gathered the user requirements and optimized the PsyMate^TM^ application and web-based reporting tool based on importance, technological feasibility and the project phase. The PAGPs and the interprofessional project group interacted continuously during the redesign process of the PsyMate^TM^.

One co-creation session was conducted with five PAGPs, their team leader and the mental health care manager of the regional primary care group using empathy mapping (Ferreira et al., 2015) to collect key needs and suggestions concerning the content and functionalities of the PsyMate^TM^ application. Empathy mapping is used to get a deeper insight into the end-users’ characteristics and their needs. In advance of the session, participants were required to use the PsyMate^TM^ application. Having an intake patient with a lifestyle problem in mind, PAGPs were encouraged to articulate what this patient would ‘say,’ ‘think,’ ‘do’ and ‘feel.’ During the discussion of the empathy map, participants focused on the match between the current version of the PsyMate^TM^ application and the required content and functionalities for the specific intake case. Next, a second co-creation session was performed with five PAGPs, their team leader and the mental health care manager using moderated user testing (Rubin and Chisnell, 2008) to collect key needs and suggestions concerning the content and functionalities of the PsyMate^TM^ web-based reporting tool. Moderated user testing requires active monitoring by a trainer to guide participants through tasks and reply to their questions, in real time. During a live demo, different views of the web-based reporting tool were demonstrated and PAGPs were invited to share their feedback. Qualitative data were recorded and transcribed verbatim. This qualitative data was analyzed by means of conventional content analysis (Hsieh and Shannon, 2005), using the qualitative analysis software Nvivo 12 for Windows. Conventional content analysis is used when literature about the topic is lacking. Predetermined categories are avoided and categories flow from the data. Therefore, the content analysis started with open coding and was followed by axial coding to group codes into categories (Hsieh and Shannon, 2005). Two researchers (ND, LH) repeatedly iterated the process of reading transcripts and organized data into groups and categories via open and axial coding. Outcomes were discussed and consensus was reached for the final thematic structure. As a result of both co-creation sessions with the PAGPs, the list of requirements was complemented. Finally, one co-creation session was executed with the interprofessional project group using the MoSCoW method (Vestola, 2010) to prioritize the user requirements and suggestions. The MoSCoW method is a requirement prioritization technique to get common understanding with different stakeholders about the importance of each requirement. Based on the importance and technological feasibility, all requirements were divided into must haves, should haves, could haves and would haves. This prioritization gave input for the software and design experts to make the first adjustments and develop a medium-fidelity prototype of both the PsyMate^TM^ application and web-based reporting tool.

#### Phase III: Materialize the New Ideas and Representations

In the ‘materialize’ phase, the PAGPs field-tested and evaluated the use of the adjusted version of the PsyMate^TM^ application and web-based reporting tool in family medicine. They reported upon the barriers and facilitators of use.

Individual coaching on the job sessions with four PAGPs were conducted to support them in using the PsyMate^TM^ in daily clinical practice. This coaching included the selection of patients who could benefit from the PsyMate^TM^, the introduction of the PsyMate^TM^ to patients in the first consultation, the download and the actual use of the application by patients in between consultations, and the use of the web-based reporting tool in the second and/or subsequent consultations. Next, the PAGPs used the PsyMate^TM^ in their daily clinical practice for 4 months. To keep the PsyMate^TM^ in the spotlight and provide support in case of questions, the researcher (ND) contacted the PAGPs every 3 weeks. Moreover, to have short communication lines, a WhatsApp group was launched. Relevant information was logged. Afterward, one focus group with two PAGPs and two individual semi-structured interviews with the remaining PAGPs were performed to evaluate the barriers and facilitators of using the PsyMate^TM^ in family medicine. The content was based on the Consolidated Framework for Implementation Research (CFIR; Damschroder et al., 2009): intervention characteristics, outer setting, inner setting, individual characteristics and process, and supplemented with questions that arouse during the focus group. Table 3 provides an overview of the interview topics. Qualitative data were recorded, transcribed verbatim and analyzed using the qualitative analysis software Nvivo 12 for Windows. CFIR informed the thematic structure during the analysis.

**TABLE 3:** Interview topics regarding the redesign and evaluation of the PsyMate^TM^ in daily practice, used for the individual interviews performed in the materialize phase.

**Main topics**	**Subtopics**
Applicability	How many times did you use the PsyMate^TM^ in daily practice?
	For which problems did you use the PsyMate^TM^ in daily practice?
Experiences patients	Content
	Design
	Burden
Experience health care professionals	Individual characteristics (health care professional and patient): attitude, behavior, knowledge, personality traits, motivation, capabilities, skills, learning style
	Relation between patient and health care professional
	Process: planning, execution, reflection, evaluation
	Inner setting: financial, societal, cultural, structural, readiness to implement, peer pressure
	Intervention: content, design, quality, applicability, adaptability, usability, complexity, costs, advantages of the implementation against business as usual, burden
Improvement areas	App
	Web-based reporting tool
	Instruction

## Results

Table 1 provides an overview of the research methodology that was applied in each phase of the design thinking model.

### Phase I: Understand the User and the User’s Problems

The first phase reviewed the characteristics of patients consulting PAGPs with mental health or lifestyle problems in family medicine; on usual care as provided by PAGPs; and on the current use of eHealth in the diagnosis and treatment of these patients. According to the literature scan, eHealth is used in family medicine (1) to reach remote patients, (2) to enhance user-friendliness, (3) to improve the accessibility of care, and (4) to stimulate patient’ empowerment and independence of health care professionals (Schalken, 2013). Patients from remote regions and patients who are less mobile or have a hearing disability can be reached relatively effortlessly via the internet. eHealth allows health care professionals to tune into the world of the patient. Furthermore, it can be tailored to the individual’s needs and communication can be focused. In addition, the patients’ anonymity can be maintained. However, the challenge is to engage patients and keep them engaged for a longer time period. Therefore, content needs to be interesting, interactive and updated regularly. Using social media, gaming elements and technology to enhance privacy and external clinical services are still opportunities that need further investigation. Patients expressed their concerns about the storage and processing of their data, and both patients and health care professionals worry about the reliability and trustworthiness of the content and the devices used (Illiger et al., 2014). Consequently, health care professionals were hesitant about using eHealth during patient contact due to the technological advancements of the devices. In addition, several individual characteristics also play a role in the acceptance of the use of eHealth in family medicine. For health care professionals, only gender played a role in the use of eHealth meaning that eHealth was more often used by male health care professionals compared to female health care professionals (Illiger et al., 2014). However, age and educational level contributed to the use of eHealth for patients meaning that young and high educated patients made more use of eHealth than elderly and educationally disadvantaged patients (Illiger et al., 2014).

In the present study, the patients had psychosocial problems ranging from symptoms such as sleep and self-esteem problems to disorders such as anxiety and depressive disorders. According to the PAGPs, the most common problems were anxiety and panic disorders, burnout, depressive disorders, sleep problems and self-esteem. They reported that moment-to-moment assessment might be useful to clarify complaints in patients with vague symptoms. eHealth modules^2^ were already used on a small scale by the PAGPs. Applications were implemented for diagnostic, psycho-educative and intervention purposes. The PAGPs noted that the intervention modules are useful, however, the diary modules, designed to collect assessment data, are insufficiently structured and transparent to interpret during patient consultations. The link between diary assessment and interventions is poor. Monitoring tools are often used as home assignments. However, the collected data often remains underused. They seem a separate element. Linking diary data to an intervention is a challenge.

### Phase II: Explore New Ideas and Representations

The second phase targeted the content and functionalities of the current PsyMate^TM^ application and web-based reporting tool and took into account suggestions for improvement from the PAGPs. Based on the prioritization of the suggested improvement areas, reviewed by the interprofessional project group, a medium-fidelity prototype was developed.

**FIGURE 3:**
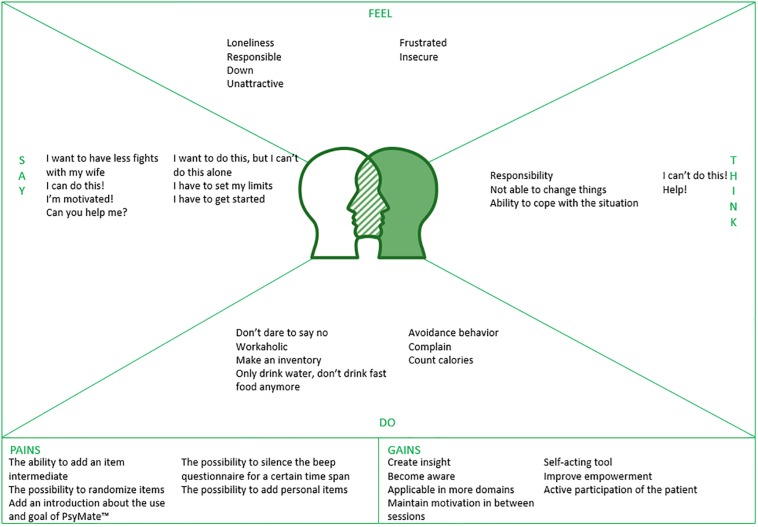
An empathy map for a patient with a lifestyle problem during a consult with a psychological assistant to the general practitioner.

Empathy mapping provided insight into what a patient with a lifestyle problem (i.e., emotion regulation problems, disordered eating behavior, reduce alcohol use and stress related problems) would say, think, do and feel during a first consultation with a PAGP. The empathy map is depicted in Figure 3. One example was a woman with overweight who feels down and unattractive. She thinks: “I will never lose weight. I better don’t say that I also experience regular eating binges. The PAGP is rather slim.” She only drinks water, runs four times a week, does not eat pie anymore and cancels birthday parties. She says that she wants to lose 25 kilos in 6 months. She is really motivated and asks the PAGP to help her achieve her goal. When comparing these ideas with the current content of the application, information that a patient is willing to share – either explicit or implicit – was mainly covered by the beep, morning and evening questionnaires. Suggestions for improvement included the incorporation of clear and concise items; the space to add personal items; the opportunity to silence the beep questionnaire for a certain time span; and this all within an intuitive and easy-to-use tool with a visual feedback mechanism, preferably within the application.

Moderated user testing yielded feedback on the web-based reporting tool. In an interactive setting, the PAGPs asked to clarify how to read (e.g., the meaning of different line graphs, symbols, colors and values), interpret [e.g., single (e.g., alone) and combined (e.g., alone at home) values, over various time spans] and explain (e.g., all the information to patients, within a short consultation) the web-based reporting tool. For patients to be encouraged and stay motivated, adequate instructions with a focus on both vulnerabilities and strengths in their daily life – in line with the concept of positive health – were proposed as important references during the implementation. Concerns were raised that less educated or skilled patients would have trouble understanding the web-based reporting tool. For that reason, suggestions for the redesign included one average line for all items, the use of smiley faces, the use of pie charts instead of line charts, and more descriptive information about the meaning of values on the axes of the charts. After all, the PAGPs also agreed that the PsyMate^TM^ was insufficiently used to really be able to assess before redesigning.

*“The web-based reporting tool can be super clear in the end, at least if this content and these functionalities stay and eventually it should be fine-tuned once. But fine-tuning is not even necessary now. You have a lot of information that can be very useful, especially in our job because we cannot dig much further.”* (Psychological assistant to the general practitioner C, moderated user testing, concluding feedback on the web-based reporting tool).

The interprofessional project group prioritized all requirements from the first two phases and weighted the importance of the user requirements to improve usability and technical difficulties to make the changes. The MoSCoW template is shown in Table 4. Based on this prioritization, the application was optimized with an option to (de)activate five personal items in the beep questionnaire and the possibility to silence the beep signal for a certain time span.

**TABLE 4:** User requirements for the PsyMate^TM^ application and web-based reporting tool subdivided within the MoSCoW template.

	**Must haves**	**Should haves**	**Could haves**	**Would haves**
PsyMate^TM^ application	– add reward gamification elements – create insight into the progress of the beeps (in-app feedback) – develop an explanimation or a manual – create a personal profile	– add a memo button	– the ability to randomize items – add a menu button – add personal items intermediate	– adjust the frequency of the beeps – adjust the time period – add goal setting elements – add an e-community function
PsyMate^TM^ web-based reporting tool	– develop a manual – add a reset button – create a simple and advanced web-based reporting tool	– make the completed memos visible	– add a menu button – create a personal profile	– make notes – add a chat function to keep in contact with the health care professional

### Phase III: Materialize the New Ideas and Representations

The third phase assessed the usability and feasibility of the optimized version of the PsyMate^TM^ application and web-based reporting tool for daily clinical practice by PAGPs. The results were structured according to the five constructs of the Consolidated Framework for Implementation Research (CFIR; Damschroder et al., 2009): intervention characteristics, outer setting, inner setting, individual characteristics and process.

#### Intervention Characteristics

The first domain of CFIR is related to key attributes of the innovation – in this case the PsyMate^TM^ application and web-based reporting tool – and includes for instance the advantage of the intervention compared to an alternative, the complexity of the intervention or the ability to test the intervention on a small scale. The PAGPs had difficulties applying the newly developed prototype PsyMate^TM^ in daily clinical practice. Consequently, they barely used it during their consultations with patients. According to database logs, only 15 patients signed up for the PsyMate^TM^ during the 4-month pilot period. This was confirmed during the three weekly telephone conversations. The PsyMate^TM^ proved to be too complex and disruptive for the highly structured and time-limited work processes in family medicine. As a consequence, effects as well as (dis)advantages of the tool were not evident. Due to the minimalistic implementation, the focus group and the interviews could not confirm that the PsyMate^TM^ contributed to more in-depth consultations or more insight into the contextual variation of mental states (functional analysis) by PAGPs and patients. The PAGPs lack the knowledge and skills to use the PsyMate^TM^ as a tool to support their functional analysis. Furthermore, the web-based reporting tool was insufficiently intuitive. However, compared to another eHealth application, the moment-to-moment assessment approach of the PsyMate^TM^ was experienced as more valuable for assessment, but the PAGPs required more help to make the link to interventions. The use of the PsyMate^TM^ could be considered both a technological as well as a behavioral innovation.

*“PsyMate^*TM*^ is now seen as a diagnostic tool. It is fairly clear for whom we should not use the PsyMate^*TM*^*. *However, we often do not realize when we can use it. When it is possible, we do not think enough about it, we will not make the connection with the PsyMate^*TM*^ as a tool for detailed functional analysis.”* (Psychological assistant to the general practitioner C, focus group, evaluation of the use of the PsyMate^*TM*^ after a 4-month pilot).

#### Inner and Outer Setting

The next two domains of CFIR apply to the inner and outer setting of the innovation – in this case the regional primary care group and the general practices – and includes among others the internal architecture of the organization and the innovation climate as well as external policies and incentives. The PAGPs have only 30 min for every patient. This made it difficult to properly introduce the PsyMate^TM^, explain the rationale, provide instructions and discuss collected individual data – aside from all regular consultation topics. In addition, the regional primary care group is a national leader in the implementation of different primary care innovations (e.g., positive health, eHealth). Under the ‘Blue Care’ (Blauwe Zorg) label, they build innovation networks with regional somatic and mental health specialist resources, health insurers and municipalities. They stimulate eHealth and other innovations. Furthermore, the general practitioners were not involved in the training and briefings. They only gave consent to the PAGP to use the PsyMate^TM^ with their patients as the implementation of an innovation in daily clinical practice. The PsyMate^TM^ was not a registered intervention with a specific financial backing and the PAGPs had to implement it in their regular consultations. From a research point of view, the PAGPs were the participants of the research and patients did not give consent. Consequently, patient experiences could not be included. The PAGPs reported that patients find it important to have a goal to monitor progress and visualize outcomes. Furthermore, the PAGPs quickly continued with their issues of the day, therefore, forgot to embed the PsyMate^TM^ during the consultations with their patients.

*“Apparently, we must be competitive with everything that is already there, but is that true, should we go that far? We must find a balance between going to the PAGP for health and rewarding people for it. We must be careful that it will not become a mass culture. It must remain professional of course.”* (Psychological assistant to the general practitioner A, focus group, experienced peer pressure).

#### Individual Characteristics

The fourth domain of CFIR concerns the characteristics of the individuals – in this case the PAGPs – and includes for example one’s knowledge, beliefs and self-efficacy regarding the intervention. The PAGPs who were interested in using eHealth in their daily clinical practice participated voluntarily in this study. Consequently, probably only the early adopters were included. The PAGPs saw face-to-face contact as the means to collect information. eHealth tools were seen as an addition to the regular treatment instead of as a replacement for the regular treatment. So, implementing tools such as the PsyMate^TM^ requires a mind shift in the way of working. Furthermore, when the PsyMate^TM^ was introduced, it was during the first or the second consultation. During subsequent consultations, the collected individual data was discussed via the web-based reporting tool. Afterward, the use of the PsyMate^TM^ faded. The PAGPs had trouble keeping their patients motivated for a prolonged time period. Ideally, 100 observations are needed for a rich functional analysis. Overall, the PAGPs considered themselves incapable of using the PsyMate^TM^ during their consultations with patients, however, they remained enthusiast.

*“It should come naturally. I am struggling to embed the PsyMate^*TM*^ in my current working routine, but that is because I think it is important to pay attention to the patients when we have a consultation. The assumption is that I do not pay attention to them when I use the PsyMate^*TM*^ and MindDistrict, although I know it has added value.”* (Psychological assistant to the general practitioner C, focus group, embedding innovative tools in daily clinical practice).

#### Process

The fifth and final domain of CFIR is the implementation process and includes planning, engaging, executing, reflecting and evaluating. Although the PAGPs agreed upon the added value of the three-weekly telephone contact with the researcher and the WhatsApp group, coaching did not emphasize the use of the PsyMate^TM^ as a tool for detailed functional analysis enough. In addition, the research team assumed the relevance of understanding symptom variation (functional analysis) was self-evident and no use cases were provided. In practice, the PAGPs were not accustomed to do this and missed hand-on instructions for the use of the PsyMate^TM^ (e.g., introduction and feedback) in daily clinical practice. The PAGPs were rather provided with concrete work instructions by means of a manual and an instruction card. Moreover, the focus group and individual semi-structured interviews, in which the PAGPs shared their experiences and reflected on their own and each other’s way of working, led to new insights and a different way of introducing and using the PsyMate^TM^.

*“We should keep using the PsyMate^*TM*^ and then we will see the value again. We should stay alert. Emails will disappear, however, we cannot get out of it when we have to answer a call.”* (Psychological assistant to the general practitioner A, focus group, the importance of keeping them engaged during the whole study).

## Discussion

### Principal Results

The aim of the current study was to investigate the redesign process of the ESM application PsyMate^TM^ as a tool for detailed functional analysis and disease management used by PAGPs in family medicine. The redesign process was based on a design thinking framework and consisted of three phases: ‘understand,’ ‘explore’ and ‘materialize.’ It disclosed essential insights into the implementation barriers and facilitators in the specific context and procedures of family medicine.

First, end-user’s characteristics were explored. Patients with psychosocial problems in family medicine are a heterogeneous group, including sleep and self-esteem problems, panic and depressive disorders or vague and unclear symptoms. Because of this diversity and in line with the emerging concept of positive health, it is important to focus on both vulnerabilities and resilience (strengths) and take transdiagnostic assessment into account (Huber et al., 2011; van Os et al., 2019).

Second, users required an intuitive, easy-to-use monitoring tool with clear and concise items and data visualization, preferably within the application. The PAGPs were satisfied with the item content, but suggested improvements for (motivational) functionalities and instructions. A medium-fidelity PsyMate^TM^ prototype was developed based on importance and technological feasibility. This matches criteria from previous research on engagement and satisfaction with mHealth applications: easily accessible and easy-to-use self-monitoring tools with an attractive user interface and tailored to the individual (Sama et al., 2014; Tang et al., 2015).

Finally, the PAGPs had difficulties using the PsyMate^TM^ in their daily clinical practice. The most important problems were: (1) there was insufficient time per consultation to properly introduce the PsyMate^TM^ and discuss the results (inner setting); (2) they felt incapable and lacked skills and knowledge to use the PsyMate^TM^ as a functional analysis tool during consultations (individual); and (3) the web-based reporting tool proved difficult to understand (intervention). The underlying problem for the PAGPs was, however, the required mind set to shift from assessing stable diagnoses toward interest in momentary variations in mental state and context that reflect vulnerabilities as well as resilience (strengths). This shift proved difficult to realize, even though the PAGPs were trained in positive health. In addition, it is important to keep in mind that eHealth and ESM are not suitable for every patient. Some patients are not able to fill in repeated assessments due to regulations in the work place. Other patients do not want to gain insight into their complaints and underlying patterns or psychological mechanisms. Consequently, the big question remains how to define the adequate patient population who can benefit from using eHealth and ESM. It is important for future implementations not to assume users are prepared and to better instruct professionals in the clinical use of momentary variations in mental states for functional analysis. These bottlenecks were also identified in research on implementation of patient reported outcome measures (PROMs) (Duncan and Murray, 2012; Antunes et al., 2014; Boyce et al., 2014; Howell et al., 2015; Bantug et al., 2016; Ament et al., 2017; Greenhalgh et al., 2017; Foster et al., 2018). The studies highlighted that health care organizations should better prepare the implementation of new technologies by checking knowledge and experience of the staff and believes about the relevance of the tool, and implementing short communication lines. Also important was being able to use the data in their work, taking into account the patients’ needs, investing sufficient time and resources, and providing both theoretical and practical training.

### Strengths and Limitations

The PsyMate^TM^ was redesigned based on a needs assessment using various co-creation methods in close collaboration with the PAGPs, a regional primary care group and an interprofessional project group. Users were well-involved. Second, the PAGPs were coached by the researchers to be able to embed the PsyMate^TM^ in their working routine. Third, field notes were collected and observations and interviews were transcribed to ensure data triangulation. Several strategies were used to enhance the trustworthiness, credibility and transferability of the study. Credibility was increased using peer debriefing sessions with the researchers (Guba, 1981). Co-creation sessions were conducted by experienced interviewers who frequently checked the meaning of the answers (Kvale, 1996). To improve transferability, information about the design, setting and participants, procedure and data collection methods was documented (Kitto et al., 2008).

As limitation, only the most intrinsically motivated PAGPs participated as they had previously shown interest in using eHealth in daily clinical practice before inclusion in this study. In addition, only the PAGPs and not the patients were involved in the redesign process. However, the PAGPs expressed the patient experiences. Moreover, patient experiences are central in a future study, a series of case studies. Finally, the interprofessional project group was unable to implement all suggestions due to technological and financial constraints. Therefore, prioritization was required. It is, however, unclear whether all suggestions are equally relevant and the realization is necessary for successful implementation.

### Implications

A sustainable implementation of mHealth tools remains a challenge in clinical care. The approach and methodology of this study identified barriers and opportunities for implementing a mHealth tool in family medicine which may be relevant to organizations intending to implement mHealth tools. There are several organizational and practical requirements to optimally implement these tools. Health care organizations should pay attention to involve both health care professionals and patients early in the process. If possible, they should be part of the redesign process to assess their needs and desires, and create ownership. Sufficient time and resources are required to ensure that they are ready for the implementation. Organizations should realize that it takes time for a new tool to become part of the daily clinical routine. In this process, nothing can be taken for granted. After all, it is not only a technological change but it also includes behavioral changes that require theoretical as well as practical training.

## Conclusion

By using the design thinking framework, the redesign process revealed meaningful insights into the needs, desires and daily routines for the implementation of the PsyMate^TM^ in family medicine. The PsyMate^TM^ is a valuable tool in mental health and psychiatric research, but implementation in family medicine, with a more diffuse patient group and less consultation time per patient, requires adjustments at all levels: the inner setting, the individual and the intervention. There were severe barriers related to the use and implementation of the PsyMate^TM^ in production-driven care where health care professionals have insufficient time and protocols reduce autonomy in the working process. Then, the lack of knowledge and skills concerning the innovative tool and its utility is difficult to compensate. Future research should focus on the trialability of the use of the PsyMate^TM^ in family medicine and incorporate patient experiences.

## Data Availability Statement

The datasets generated for this study can be found on SURFDrive (https://surfdrive.surf.nl/files/index.php/s/xbcgp3k8dBvsexw).

## Ethics Statement

Ethical approval was obtained for the patient interviews from the Medical Ethics Review Committee of Zuyderland and Zuyd University of Applied Sciences. Furthermore, we adhered to the approved ethical (consent) procedures of the Medical Ethics Review Committee of Zuyderland and Zuyd University of Applied Sciences. Since the psychological assistants to the general practitioner were the research participants and patients received regular care, these procedures do not require written informed consent. However, oral informed consent was obtained from both the psychological assistants to the general practitioner and the regional primary care group. Moreover, they also provided consent for this research via mail. In addition, ethical principles that are outlined in the Dutch “Medical Research Involving Human Subjects Act” were followed throughout the redesign process.

## Author Contributions

All authors were involved in the development of the study. ND drafted the initial manuscript. All authors edited, revised, and approved the final manuscript.

## Conflict of Interest

The authors declare that the research was conducted in the absence of any commercial or financial relationships that could be construed as a potential conflict of interest.

## Figures and Tables

**FIGURE A1:**
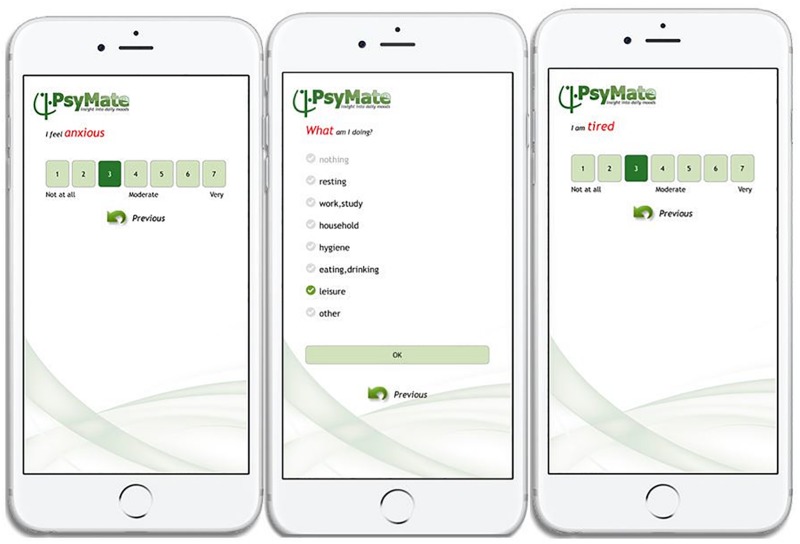
Examples of a mood, context and physical status item within the beep questionnaire.

## Footnotes

1 psymate.eu

2 www.minddistrict.com

## Appendix A

The PsyMate^TM^ users gather data for at least hundred beep moments to be able to optimally use the web-based reporting tool. The application runs on every mobile phone with Android or iOS software (Appendix Figure A1); the web-based reporting tool is accessible from every computer with Internet connection. People need personalized log in credentials to gain access to both the application and the web-based reporting tool. Monitored data is anonymously and confidentially saved on secured servers that comply with present rules and regulations. The PsyMate^TM^ can be used for different purposes: (1) as a diagnostic tool, (2) for monitoring therapy and medication, (3) for patient empowerment, and (4) prevention purposes (Palmier-Claus, 2011; Groot and van Ingen Schenau, 2013; Simons et al., 2015; Verhagen et al., 2016; Kossakowski et al., 2017; van Os et al., 2017). However, the PsyMate^TM^ is previously only investigated for research purposes in mental health, psychiatry, developmental psychology, and high specialist somatic care. Applications in family medicine were never explored. The moment-to-moment assessment of mood and behavior in the context of daily life give people insight into their daily life patterns. Mechanisms underlying their symptomatology can be charted. People are experts of their daily life. Consequently, treatment can become tailored coproductions. This fosters commitment and motivation by the patient (Boyce, 2011). Tailored coproduction can also lead to patient’ empowerment with a focus on mental resilience and an increase in positive experiences. To reduce the chance of relapse, recovered patients can use a self-management tool (Boyce, 2011).
